# Disentangling genetic vs. environmental causes of sex determination in the common frog, *Rana temporaria*

**DOI:** 10.1186/1471-2156-9-3

**Published:** 2008-01-08

**Authors:** Chikako Matsuba, Ikuo Miura, Juha Merilä

**Affiliations:** 1 Ecological Genetics Research Unit, Department of Biological and Environmental Sciences, PO Box 65, University of Helsinki, FI-00014 Helsinki, Finland; 2 Institute for Amphibian Biology, Graduate School of Science, Hiroshima University, Japan

## Abstract

**Background:**

Understanding of sex ratio dynamics in a given species requires understanding its sex determination system, as well as access for reliable tools for sex identification at different life stages. As in the case of many other amphibians, the common frogs (*Rana temporaria*) do not have well differentiated sex chromosomes, and an identification of individuals' genetic sex may be complicated by sex reversals. Here, we report results of studies shedding light on the sex determination system and sex ratio variation in this species.

**Results:**

A microsatellite locus RtSB03 was found to be sex-linked in four geographically disparate populations, suggesting male heterogamy in common frogs. However, in three other populations examined, no or little evidence for sex-linkage was detected suggesting either ongoing/recent recombination events, and/or frequent sex-reversals. Comparison of inheritance patterns of alleles in RtSB03 and phenotypic sex within sibships revealed a mixed evidence for sex-linkage: all individuals with male phenotype carried a male specific allele in one population, whereas results were more mixed in another population.

**Conclusion:**

These results make sense only if we assume that the RtSB03 locus is linked to male sex determination factor in some, but not in all common frog populations, and if phenotypic sex-reversals – for which there is earlier evidence from this species – are frequently occurring.

## Background

Most vertebrates exhibit genotypic sex determination (GSD), i.e. the sex of an individual is genetically determined. Typically, GSD involves sex chromosomes so that one of the sexes is heterogametic (XY or ZW) and the other homogametic (XX or WW). However, in some species, notably in reptiles and few amphibians, environmental sex determination (ESD) occurs [1,2]. In ESD, the sex of the individuals is determined by environmental conditions experienced during early development. Environmental factors capable of influencing sex determination include, e.g., temperature, pH, environmental contaminants, and even social conditions experienced as juveniles [1,3,4], but clearly, more experiments exploring the occurrence of environmental sex determination in amphibians is needed. However, sex determination by environmental influences occurs rarely in isolation from genetic sex determination factors, but typically also GSD is often involved. This opens a possibility for very complex sex-ratio dynamics: sex-reversed individuals (e.g. XX-males) that mate with normal females (XX) will produce only female offspring in absence of environmental influences on sex determination [1].

The sex determination system in amphibians varies from species having ZZ/ZW (e.g., *Xenopus*) to others having XY/XX systems [1,5]. Even intraspecific variation has been found [6,7]. Amphibian sex chromosomes are generally very weakly differentiated making cytogenetic sex identification difficult [8,9]. Likewise, genetic markers linked with sex determination systems are known only from a very limited number of species [7,10-13]. Consequently, it is often unclear how the sex determination in particular amphibian species functions and influences sex-ratio dynamics within and among populations.

**Figure 1:**
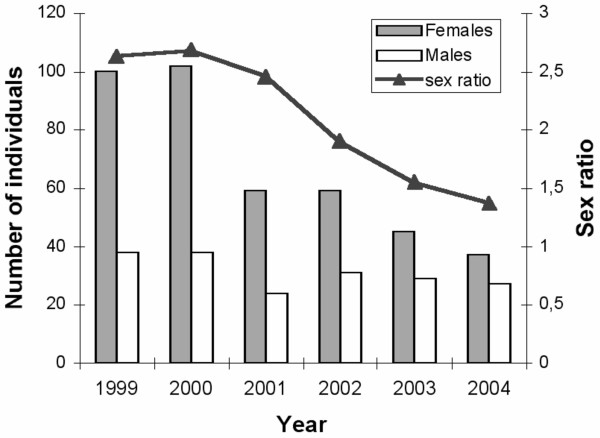
Number of male and female common frogs and corresponding (phenotypic) adult sex ratio in Kilpisjärvi study site over six years (data from [14] and [15]).

A Capture-Mark-Recapture (CMR) study of common frogs in two ponds in northern Finland has revealed peculiar sex ratio dynamics [14]. The sex ratio of breeding adults at the two ponds was strongly female-biased in years 1999–2000, declining somewhat thereafter (Fig. 1, [15]). Since the CMR-data demonstrates that the survival and recapture probabilities of the adults of both sexes were indistinguishable [14], the sex-ratio bias cannot be explained by differential survival or recapture probability of the sexes. Possible explanations for highly biased sex ratios in this particular case include strongly biased primary sex-ratios, sex differences in early life mortality, and/or sex-reversals caused by some environmental factor such as temperature [16,17]. Attempts to differentiate among these alternatives would be greatly aided by access to sex-linked markers and/or any insight on possibility that sex reversal could be taking place.

The aim of this study was to establish formally whether one particular microsatellite locus (RtSB03; [18]) in common frogs exhibit sex-linked inheritance pattern, and if so, whether this holds true in number of geographically widely separated populations. The latter question is relevant in this context as it has been suggested that there exists geographic 'races' in common frogs in respect to development of their gonads [17]. It is also relevant in the sense that if evidence for sex-linked inheritance will be found, the utility of this marker as a sex-identification tool needs to be verified in multiple populations. In order to accomplish the above mentioned goals, we looked for evidence of sex-linked inheritance RtSB03 alleles using data on adult frogs collected from the wild, as well as association between phenotypic sex and RtSB03 genotypes within full-sib families hatched from wild collected eggs.

## Results

### Identification of sex-linked microsatellite marker in samples from the wild

Initial testing of possible sex-linkage was carried out on using previously published data [19] on seven Fennoscandian populations of common frogs genotyped for eight microsatellite loci: Rt2Ca2-22, Rt2Ca25 [20], RRD590 [21], RtμH [22], Rtempμ4, Rtempμ5, Rtempμ7 [23] and RtSB03 [18]. To identify potentially sexed-linked markers we tested all loci for deviations from Hardy-Weinberg equilibrium (HWE) in pooled samples. This initial screening revealed that one of the markers – RtSB03 – deviated from HWE in four out of the seven populations in the two tests (Table 1; see below for details of tests).

**Table 1:** Tests for deviations from Hardy-Weinberg expectations among wild collected adult common frogs at eight microsatellite loci. Shown are fixation indices (F_IS_) and associated significance levels (F) as well as significance levels from heterozygosity excess tests from Genepop program (*G*). Bold stars indicate the adjusted significance level (5%) after Bonferroni correction at population level. Data from ref. [33]

		RRD590			RtCa2			RtCa25			RtGR04			RtGR05			RtGR07			RtH			RtSB03
		
Population	Coordinates	F_IS_	*F*	*G*	F_IS_	*F*	*G*	F_IS_	*F*	*G*	F_IS_	*F*	*G*	F_IS_	*F*	*G*	F_IS_	*F*	*G*	F_IS_	*F*	*G*	F_IS_	*F*	*G*
Kilpisjärvi	69°03'N, 20°07'E	-0.058			-0.014			0.111			-0.037			-0.253	*	*	-0.020			0.099			-0.258	**	**
Kiruna	67°01'N, 21°00'E	0.098			-0.209			0.075			-0.086			-0.024			-0.055			0.218			-0.069		
Ammarnäs	65°54'N, 16°18'E	-0.016			-0.216			0.072			-0.245	*	**	-0.104			0.123			-0.195	*	*	-0.309	**	
Hamptjärn	63°52'N, 20°03'E	0.039			-0.176			0.167			-0.150			-0.132			-0.088			0.099			0.002		
Grytan	63°49'N, 20°04'E	0.17			-0.129			-0.033			-0.145	°		-0.127		*	0.117			0.068			-0.150	*	**
Häggedal	59°51'N, 17°14'E	0.122			-0.093			0.162			0.024			-0.211	*	*	-0.067			0.387			-0.136	°	
Tvedöra	55°42'N, 13°26'E	0.087			-0.158			0.152			0.140			0.266			0.146			-0.090			0.090		

***p < 0.001, **p < 0.01, *p < 0.05, °p < 0.1

### Sibship sex ratios

A total 261 individuals from eight sibships, one from Helsinki and seven from Kilpisjärvi were sexed by gonadal inspection (Table 2). Five sibships from Kilpisjärvi (Kil-1, 3, 4, 7 and 8) had highly male-biased sex ratios ranging from 72 to 100% males (Table 2). In contrast, one of the Kilpisjärvi families (Kil-6) exhibited a strongly female-biased sex-ratio (χ^2^_1 _= 5.45, p < 0.025). Only the sibship from Helsinki, and one of the Kilpisjärvi (Kil-5), did not exhibit any deviations from 1:1 sex ratio (Table 2). Although some, but very low degree of mortality occurred in the sibships before sexing (x = 5.6%, Table 2), this mortality was uncorrelated with sibship sex ratios (*r *= -0.44, *n *= 8, *P *= 0.27). This suggests that the deviations from 1:1 sex-ratios are unlikely to be explained by differential mortality among sexes.

**Table 2:** Sib-ship sex-ratios based on phenotypic sexing (gonadal inspection) of 2–6 months old froglets. "Intermediate" refers to gonadal phenotype bearing characteristics of both sexes. χ^2 ^is the value of chi square statistics testing for deviations from 1:1 sex-ratio. Degree of freedom is 1 for all tests. % mortality refers to percentage of mortality in each family that occurred before sexing

Sib-ship ID	Total	Females	Males	Intermediate	% mortality	Sex-ratio	χ^2^	*p*
Helsinki	49	18	31	0	5.6	0.63	3.45	
Kil-1	28	3	25	0	10.0	0.89	17.28	***
Kil-3	36	8	27	1	3.6	0.77	10.31	***
Kil-4	23	0	23	0	1.8	1.00	23	***
Kil-5	33	15	18	0	2.5	0.55	0.27	
Kil-6	31	22	9	0	8.9	0.29	5.45	*
Kil-7	43	12	31	0	6	0.72	8.39	***
Kil-8	18	1	17	0	0	0.94	14.22	***

***p < 0.001, *p < 0.05

### Sibship genotypes

Five sibships (Helsinki, Kil-3, 5, 6 and 7) consisting altogether of 158 individuals with sufficient number of representatives in both sexes were genotyped for the RtSB03 locus (Table 3). The Helsinki sibship (n = 23) had three alleles and there was evidence for sex-linkage: the 07-allele was found only in male offspring, and hence, probably came from their father (Table 3). Also a significant association with alleles and sex was indicated by the trend test (χ^2 ^= 23, df = 2, *p *< 0.001). The Kil-7 family (n = 41) had four alleles and clear male-bias in the distribution of allele 09. This allele seems to be male specific in wild collected Kilpisjärvi individuals too, although a small proportion of females appear to carry it as well (see below). The trend test could not be performed in this sibship because the variance-covariance matrix was singular. However, no females carried this allele in this sibship, and some males also did not carry this allele (Table 3). Unfortunately, three of the four Kilpisjärvi sibships were mono- or bi- allelic, and could not be analyzed for marker-sex associations.

**Table 3:** Observed genotypes of RtSB03 locus and individual number for each genotype in sib families. Parent genotypes were predicted from sibship genotypes and their frequency. n = number of individuals

			Sibship genotype				Predicted parent genotype
			
Sib family		n	(04/04)	(10b/04)	(04/07)	(10b/07)	(04/10b)*(04/07)
Helsinki	Total	23					
	Female	13	8	5	0	0	
	Male	10	0	0	5	5	

			(09/13)				(09/09)*(13/13)

Kil-3	Total	33	33				
	Female	8	8				
	Male	25	25				

			(13/13)				(13/13)*(13/13)

Kil-5	Total	32	32				
	Female	14	14				
	Male	18	18				

			(04/04)	(04/13)			(04/04)*(04/13)

Kil-6	Total	29	16	13			
	Female	20	13	7			
	Male	9	3	6			

			(04/11)	(13/11)	(04/09)	(13/09)	(04/13)*(09/11)

Kil-7	Total	41					
	Female	12	4	8	0	0	
	Male	29	8	3	6	12	

***p < 0.001, *p < 0.05

### Linkage of sex and SB03 locus in wild population

Since the distribution of the two sexes in Palo *et al.*[19] samples (in Table 1) were unknown, we augmented samples with even sex ratio (n_males_= 124 ; n_females _= 114) by genotyping of RtSB03 locus for six populations using samples collected at same time for the same populations as the previously genotyped samples (Table 4). In the genotype data consisting of 238 adult frogs sampled from six populations, a total of 17 alleles were detected in the RtSBO3 locus (Table 4). A significant deviation from HWE within pooled sex samples was detected in two (28%) of the populations (Kiruna and Ammarnäs; Table 4). A significant deviation from HWE in males was observed in Kilpisjärvi and Ammarnäs populations in both of the heterozygosity excess tests, the test based on randomization, as well the test based on Markov chain method (Table 4). None of the tests for females revealed evidence for heterozygote excess (Table 4).

**Table 4:** Test for deviations from HWE for each sex and trend tests between sexes and alleles in RtSB03 locus in wild populations. Shown are fixation indices (F_IS_) and significance levels for heterozygosity excess tests as estimated after 12000 randomisations in FSTAT (F) and after 1000 iterations per patches of the Markov chain algorithm in Genepop program (G). df = degrees of freedom.*n *= number of individuals

			HWE deviation			Trend		
				
Population	Group	*n*	F_IS_	*F*	*G*	χ^2^	df	*p*
Kilpisjärvi	Total	34	-0.063			22.06	8	**
	Female	18	0.089					
	Male	16	-0.420	***	***			
Kiruna	Total	48	-0.101	*		14.40	10	
	Female	24	-0.138					
	Male	24	-0.071					
Ammarnäs	Total	41	-0.196	°	*	37.74	4	***
	Female	18	0.145					
	Male	23	-0.770	***	***			
Grytan	Total	40	0.407			23.63	7	**
	Female	18	0.533					
	Male	22	0.148					
Häggedal	Total	38	0.241			13.33	8	
	Female	18	0.115					
	Male	20	0.337					
Tvedöra	Total	37	0.223			8.47	10	
	Female	18	0.103					
	Male	19	0.361					

***p < 0.001, **p < 0.01, *p < 0.05, °p < 0.1

Armitage's [24] trend tests revealed significant associations between RtSBO3 alleles and sex in three (Kilpisjärvi, Ammarnäs and Grytan) of the six populations (Table 4). A closer examination of allele distributions reveled that alleles 07 (Grytan) and 09 (Kilpisjärvi, Grytan and Ammarnäs) are strongly linked to male phenotypes (Table 5). In other words, all males within these populations had at least one of these alleles, and the frequency of these alleles in females was either zero or very low (f < 0.11; Table 5).

**Table 5:** Male biased alleles and number of males having a specific allele

Population	Allele	Male	Female
Kilpisjärvi	**09**	16	4
	(total)	(16)	(18)
		100%	22%

Ammarnäs	**09**	23	0
	(total)	(23)	(18)
		100%	0%

Grytan	**07**	16	1
	**09**	8	2
	(total)	(22)	(18)
		109.1%*	16.7%

* because of one 0709 male

## Discussion

Several lines of evidence suggest that the microsatellite locus RtSB03 is sex-linked in the common frog, supporting earlier suggestions [9,16] that the sex-determination system in this species is based on male heterogamy. First, among the wild collected adults, four out of six populations displayed significant heterozygote excess in RtSB03 and these deviations from HWE were mainly due to excess of heterozygosity among males. Second, we found strong evidence for male specific alleles at least three of these populations. The results from the analyses of sibships largely corroborated with these results, although some deviations from broad patterns were evident. In what follows, will discuss these findings and interpretations, as well as some of the apparent inconsistencies in the data.

Perhaps the most convincing evidence for sex-linkage in our study comes from the segregation analysis of RtSB03 alleles in the sibship from Helsinki. All males in this family carried one copy of the same (07) allele that was absent in their female sibs. This same allele was also over-represented in males in one of the Swedish populations (Grytan), and suggesting strong linkage to sex determination locus. Unfortunately, most of the sibships from the Kilpisjärvi turned out to be uninformative for segregation analyses, either because of strongly biased sex-ratios (see below) or because of high degree of homozygosity in RtSBO3 locus in these families. Nevertheless, one of the families (Kil-7) provided clear evidence for sex-linked inheritance: none of the females carried the '09' allele in this family, while 62.1% males did. Since this allele was over-represented among adult males in this population, and found solely from males in other populations (e.g. Ammarnäs), this provides additional evidence for sex-linkage. The fact that 37.9% of males did not carry this allele in Kil-7 family could be explained in two different ways. First, the males not carrying this allele could be sex-reversed males (i.e. genetic females). Second, the absence of 09-alelle in some males could evidence for recombination among RtSB03 and sex determining locus. However, the latter explanation seems unlikely if we consider the overall inheritance patterns of alleles in this locus and family. If we assume that the male parent of this sibship had the RtSB03 genotype 09/11 (cf. Table 3), then both alleles should be produced in equal numbers in crossing-over. If the alleles are unlinked to sex determination factor, we would expect to see also the allele 09 present among female offspring. As shown, this was not case. Likewise, if eight males with 04/11 genotypes and three males of 13/11 genotypes in Kil-7 were counted as females, the sex ratio (23 females and 18 males) would fall in a range of 1:1 sex ratio (*p *= 0.43), supporting sex-reversal from genetic females to males. Hence, we conclude that the overall evidence discussed above is consistent with the interpretation that the locus RtSB03 is sex-linked, but the sex determination may also be affected by environmental factors.

Assuming that the 09-allele is linked to Y chromosome in Kilpisjärvi population, there are several exceptions to be explained. First, although we found significant heterozygosity excess in RtSB03 in Kilpisjärvi among adult males which all carried 09-allele, also 22% of females had this allele. Second, all the females in one Kilpisjärvi sibship (Kil-3) possessed the 09-allele. Third, in many families (Kil-5, 6, 7), males did not have this allele.

**Figure 2:**
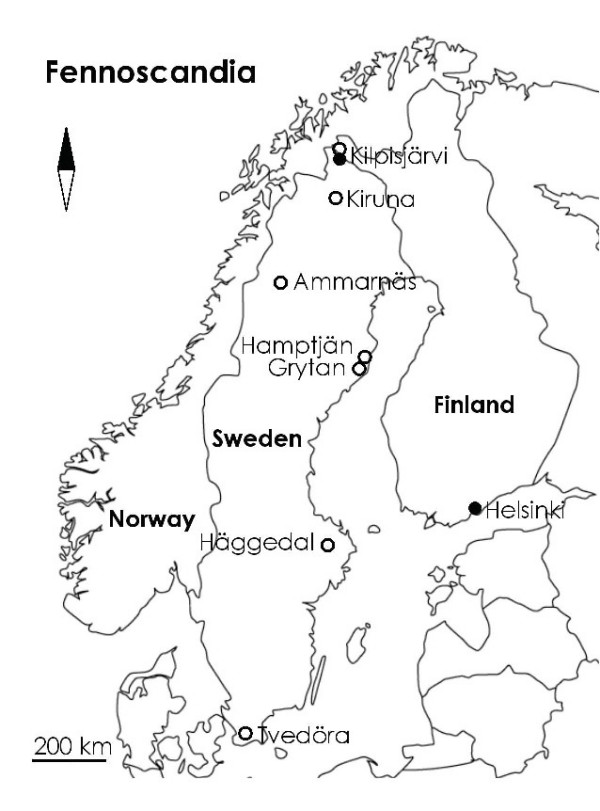
A map showing the location of the populations used in this study. Open circles depict origin of wild collected adults, and closed circles origin of sibships collected as embryos.

As suggested above, one possible explanation for these observations reside on possible sex-reversals. For instance, the sex ratio in the Kil-3 family was strongly male biased (25 males : 8 females), and all individuals had the same RtSB03 genotype (09/13). Since all individuals with this genotype should be genetically males, the eight females could be sex-reversed males (i.e. XY-females). Indirect support for this interpretation is provided by the observation that sex-ratio of adult frogs in the Kilpisjärvi population has been changing from female bias towards more even sex-ratio over the past five years (Fig. 2). In other words, if the initial distortion in the sex ratio was caused by feminization of genetic males to sex-reversed males (XY-females) by some environmental effects, mating between XY-females and XY-males is expected to produce offspring sex ratios of 1:3 (female [XX]/male [XY & YY]) or 1:2 (if YY males are sterile/unviable). Consequently, exactly as it is observed (Fig. 2), the sex ratio in the population should approach to the equal sex ratio and, eventually, turn towards male bias. Also the predicted genotype (09/09 i.e. YY-male) of the father of Kil-3 sibship supports this interpretation. Once again, recombination is unlikely to explain the relative high frequency (11.8%) of 09-females, because of the low recombination rate (0%) observed in the Kil-7 sibship.

One puzzling observation was that some of the males were missing the male specific 09-allele in sibships (Kil-5, 6 and 7) while all field collected adult males in Kilpisjärvi carry it. Again, this might be explained by sex reversals from female to male. In mammals with XY sex determination system, sex reversals from male to female (XY female) are more common than reversals from female to male (XX male). XY females are often caused by dosage-sensitive sex reversal (DSS), such as mutations on testis determining gene *SRY *on Y-chromosome [25,26], over-expression of *AHC *on X-chromosome [27], or under-expression of the autosomal genes such as *SOX9 *[28], *WT1 *[29,30] and *SF-1 *[31] by mutation/deletions. For the XX male, in a human case the translocation of *SRY *gene from Y to X chromosome caused to masculinized phenotype of XX type [32]. If this is true in frogs too, sex-reversed (XX) males (i.e. lacking 09-allele) may be rare in the wild.

Why was the sex linkage not observed in all study populations? One possible explanation is the existence of multiple sex determination loci and their weak linkage with each other. Allozyme linkage analyses of several Ranid species have suggested existence of multiple sex determination factors that are situated in different linkage groups [33]. This has been also suggested to be an explanation for undifferentiated sex chromosomes in many amphibian species [33]. In fact, some Ranid species are known to have different sex determination systems in different populations of the same species [7,33]. In other words, sex linkage in particular locus can be detected in some but not necessarily in all populations of the given species.

Finally, it is worth noticing that the sex ratios of most sibships from Kilpisjärvi deviated significantly from 1:1 expectation, being usually strongly male biased. At proximate level, such bias might arise for either genetic or environmental reasons. Since the associations between RtSB03 genotypes and offspring sex ratios were weak, this might suggest that some environmental factors have affected sex development of the sibships. Many environmental factors, such as high temperatures and hormonally acting contaminants [1,8,16,17] may induce sex reversal in amphibians. For instance, Witschi [16] has demonstrated that higher temperatures (32°C) can induce females to develop males in *Rana *frogs. Although the temperatures under which the sibships in our study were reared (ca. 20°C) did not lead sex ratio distortions in Witschi 's [16] studies, it is still possible that +20°C for sibships originating from northern Finland represent higher temperatures than these frogs would normally encounter.

## Conclusion

In conclusion, our results suggest that microsatellite locus RtSB03 is linked to male sex determination locus at least in some, but not necessarily in all, common frog populations. The observed linkage is consistent with earlier evidence for male heterogamy in species [9,17]. However, the results are also consistent with interpretation that environmental factors might be influencing sex determination in this species, making molecular sex-identification unreliable. Yet, conclusive demonstration of sex-reversals would require either controlled experiments manipulating factors that might affect sex determination and/or further work identifying candidate genes for sex determination, e.g., with genome walking around RtSB03 locus. However, the latter approach will work only if the sex determination factor(s) are situated strictly in sex chromosomes. The possibility that sex determination system in this species consists on multiple factors situated in different chromosomes cannot be currently ruled out.

## Methods

### Study species and populations

The common frog is wide spread anuran amphibian of Europe which has been subject to numerous ecological [34-37], evolutionary [37] and genetic studies [19,38,39]. The material for this study originated from seven Fennoscandian populations as shown in Fig. 2. The adult samples were collected in 1998–2001 as part of earlier studies [19,20,40], while the sibships from Helsinki (n = 1) and Kilpisjärvi (n = 8) were collected in April and June 2005, respectively. All of the frogs used in this study were collected from breeding sites at peak of the breeding activities, and the phenotypic sex of adults was determined by dissection of the gonads in laboratory.

DNA from these populations for genotyping of RtSB03 was extracted by the glass fiber filter method [41]. For further details of data collection, see Palo *et al.*[19,20,40].

### Sibship material

To test for correspondence between phenotypic sex and segregation of RtSB03 alleles we collected approximately 100–200 fertilized embryos per clutch from one clutch in Helsinki (60°10' N, 24°41' E) and eight clutches at two breeding sites in Kilpisjärvi (69°02' N, 20°49' E and 69°03' N, 20°45' E). Rearing of the sibships as well as their phenotypic sexing took place in Hiroshima (Japan) where the collected embryos were shipped by air directly after collection. These embryos were reared through metamorphosis (at ca. 20°C) in the laboratory until they had completed their gonadal development. To assess the completion of the gonadal development, we sampled the sibships during several time points (viz. two weeks, one-two months and five-six months after metamorphosis) and examined the stage of gonadal development by dissection [17]. One month after metamorphosis, six out of 89 individuals had not yet completed their gonad development, but after two months all but one out of 261 examined frogs had completed their gonadal development. Therefore, it was decided that phenotypic sexing of sibships was conducted two to six months after metamorphosis. At the time of the phenotypic sexing, a liver sample was collected from all froglets to be used in DNA extraction. The sex ratio of each clutch was tested for a deviation from 1:1 sex ratio using chi square goodness of fit tests.

Since the sibships originated from wild collected clutches, the parental genotypes were not known. This is potentially problematic because multiple paternity is know to occur in common frogs [42], and hence, might cause problems in interpretations. However, as it turned out, the maximum number alleles found from each of the five genotyped sibships was four, suggesting that multiple paternity did not occur in sibships used in this study.

### Genotyping on RtSB03 locus

Genotyping of RtSB03 both on adults and metamorphs was carried out with primers SB03-A2(21) (5'-ATG CAC AGC TCT TTG ACT CTC-3') and SB03-BtailFAM (5'-GTTTCCACTGCGATTCTGACCTGTG-3'; modified from [18]). PCR reactions were prepared in total volume of 10 μl (ca. 20 ng of template DNA, 1 μM FW and RV primer, 1.5 mM Mg_2_Cl, 0.25 mM dNAPs, 1× reaction buffer and Taq DNA polymerase) and subjected to 35 amplification cycles (94°C for 30 sec, 50°C for 30 sec and 72°C for 30 sec) and one extension step at 72°C for 5 min. The PCR products were separated using the Megabace 1000 capillary sequencer (Amersham Bioscience), and signal peaks were analyzed with the Peakprofiler program (Amersham BioScience). To ensure accuracy of the genotype data, we genotyped 48 samples twice for the RtSB03-locus to estimate the genotyping error rate. We did not find any genotyping errors. The low frequency of genotyping errors is also suggested by the fact in the sibship data (n = 158 genotypes), we never found any alleles that would have been inconsistent with inheritance patterns expected for bi-allelic loci.

### Allele frequency tests

The logic for detecting sex-linkage was as follows. If the target locus is situated in the sex chromosomes and the allele(s) in the minor chromosome (Y or W) is not shared with the major (sex) chromosome (X or Z), the observed frequency of heterozygotes in the pooled sex sample should be higher than that expected under Hardy-Weinberg equilibrium (HWE), and especially so in the heterogametic sex [12,13]. Furthermore, marker situated on Y or W chromosome should also be less variable than that those in autosomes because of lack of recombination. Therefore, expected hetero- and homozygosities were calculated for three groupings of wild caught individuals: pooled sexes, females and males. Three tests of heterozygosity excess were employed. First, F_IS _index [43] was calculated using the FSTAT program v.2.9.3 [44] and tested for difference from zero using a randomisation procedure within FSTAT. Second, heterozygote excess was also tested using the Score (*U*) test with Genepop v. 3.4 [45] where the significance is estimated by the Markov chain method [46]. Third, we tested for an association between phenotypic sex and RtSB03 alleles using the trend test [47] which is a score test for binominal traits (in this case sexes [24]). The trend test is suitable in the cases where the loci are not in HWE [47,48] and it was done using the program PowerMarker [49].

## Authors' contributions

CM carried out the sampling and the molecular genetic work, performed statistical tests, and drafted the manuscript. IM carried out the preparation of samples and the inspection of sex, and helped to draft the manuscript. JM carried out the initial sampling, conceived of the study and constructed the final version of manuscript. All authors have read and approved the final manuscript.
